# Subtyping *Salmonella* isolated from pet dogs with multilocus sequence typing (MLST) and clustered regularly interspaced short palindromic repeats (CRISPRs)

**DOI:** 10.1186/s13568-021-01221-9

**Published:** 2021-04-24

**Authors:** Cheng Yang, Wangfeng Shao, Lingling Wei, Lingxiao Chen, Aihua Zhu, Zhiming Pan

**Affiliations:** 1 College of Health Sciences, Jiangsu Normal University, Xuzhou, 221116 Jiangsu China; 2 School of Life Sciences, Jiangsu Normal University, Xuzhou, 221116 Jiangsu China; 3 Jiangsu Co-Innovationnovation Center for Prevention and Control of Important Animal Infectious Diseases and Zoonoses, Yangzhou University, Yangzhou, 225009 Jiangsu China; 4 College of Arts and Sciences, Suqian University, Suqian, 223800 Jiangsu China

**Keywords:** *Salmonella*, Pet, Multilocus sequence typing, Clustered regularly interspaced short palindromic repeats, Molecular subtyping

## Abstract

*Salmonella*, as a zoonotic pathogen, has attracted widespread attention worldwide, especially in the transmission between household pets and humans. Therefore, we investigated the epidemic distribution of dog *Salmonella* from pet hospitals and breeding base in Xuzhou, Jiangsu Province, China, and used multilocus sequence typing (MLST) and clustered regularly interspaced short palindromic repeats (CRISPRs) to subtype *Salmonella* isolates. From April 2018 to November 2019, a total of 469 samples were collected from pet hospitals and breeding base, including 339 dog samples and 60 cat samples. *S.* Kentucky (40.74%) was the most prevalent serotype, but other, such as *S.* Typhimurium (18.52%) and *S.* Indiana (18.52%), were also widespread. Eight different sequence type (ST) patterns were identified by MLST and ST198 was the highest proportion of these isolates. CRISPRs analysis showed that 9 different Kentucky CRISPR types (KCTs) was identified from ST198. 48 spacers including 29 (6 News) for CRISPR1 and 19 (4 News) for CRISPR2 that proved the polymorphic of *Salmonella* genes in samples from different sources. The analysis demonstrated that the common serotypes were widely present in pet hosts in the same area. This analysis shows that CRISPR genes have better recognition ability in the same serotype, which has a positive effect on the traceability of *Salmonella* and the prevention and treatment of salmonellosis.

## Introduction

*Salmonella*, as a kind of zoonotic bacteria, has caused widespread concern worldwide. In recent years, the number of pets worldwide has increased dramatically, of which cats and dogs account for 80%. The close contact between humans and pets also greatly increases the risk of *Salmonella* spreading between pets and humans. Previous investigations showed that the positive rate of *Salmonella* in pet dogs with diarrhea is more than twice that of healthy pet dogs (Renate Reimschuessel et al. 2017). To make matters worse, healthy pets with no obvious symptoms will also release *Salmonella* to the outside environment in the form of feces, causing cross-infection between humans and other animals (Wray and Wray 2000). *Salmonella* can also spread among animal groups (Van Immerseel et al. 2004). There are even individual cases that can prove that pet dogs and cats can spread their own *Salmonella* to humans (Cherry et al. 2004; Koehler et al. 2006). From 2006 to 2008, in the United States, a study related to human *Salmonella* infection and contaminated dry dog and cat food showed that *Salmonella* is more likely to spread between infants and young children (Behravesh et al. 2010). Interestingly, In southern Ontario, Canada, Murphy tested 188 dogs and 39 cats for *Salmonella*, and found no positive strains (Murphy et al. 2009). Therefore, it is necessary to pay more attention to *Salmonella* from pets and Salmonellosis prevention in China which have the largest number of pets.

The genetic characteristics of pet-derived *Salmonella* have not been extensively studied. At present, molecular detection methods are widely used in the typing of *Salmonella* strains. Especially MLST, which is more efficient and accurate than pulse field gel electrophoresis (PFGE), and is more suitable for the identification and differentiation of animal *Salmonella* (Shi et al. 2013). The results of MLST are helpful to analyze the genetic characteristics of different serotypes of *Salmonella*, and prove the existence of microevolution between different STs. On the other hand, for the same serotype, MLST could not accurately demonstrate the genetic and phenotypic differences of *Salmonella* (Tennant et al. 2010; Gymoese et al. 2017). Therefore, a new identification method was established (Fabre et al. 2012). CRISPR has a higher resolution for identical serotypes than PFGE (Thompson et al. 2018). This study investigates the genetic diversity and subtype transmission of pet *Salmonella* between pets and humans in Xuzhou, China. Two molecular typing methods were used. 27 strains of *Salmonella* from pets were identified with different subtypes using MLST; CRISPR was used to analyze the identical serotypes of *S.* Kentucky and estimate the potential risk of the same subtypes *Salmonella* spreading between pets and humans.

## Materials and methods

### Sample collection and Serotyping

From April 2018 to November 2019, 469 samples were from 8 different pet hospitals and a breeding base, of which 339 were from dogs and 130 were from cats. Rectal sampling of pets using cotton swabs infiltrated with BPW and then strictly followed the Chinese National Standard Method (GB 4789.4-2016) for *Salmonella* isolation, the serotypes of all of the strains determined by slide agglutination according to the Kauffman-White serotyping scheme with O- and H-antigen specific sera.

### Multilocus sequence typing (MLST)

The confirmed strains were cultured with LB medium at 37 °C and 120 rpm/min shaking for 18 h, Then genomic DNA was extracted with the TIANamp Bacteria DNA kit (Tiangen Biotech (Beijing) CO., LTD, China) according to the manufacturer's protocol. Each identified strain was characterized by MLST using seven housekeeping (Table 1) (Kidgell et al. 2002). PCR products were delivered to Sangon Biotech (Shanghai) Co., Ltd. for purification and sequencing, and the seven pairs of housekeeping gene testing and sequencing results of each strain were uploaded to the MLST database for comparison (http://mlst.warwick.ac.uk/mlst/dbs/ Senterica). This is used to determine the sequence type (ST) of each strain. A minimum spanning tree was generated using BioNumerics software, version 7.5 (Applied Maths, Kortrijk, Belgium) to analyze the distribution of STs from cats, dogs and humans.

**Table 1:** Sequencing and amplification primers for *Salmonella* MLST typing

Gene	Primers		Size(bp)
	Amplification primers (5′-3′)	Sequencing primers (5′-3′)	
aroC	F: CCTGGCACCTCGCGCTATAC	F: GGCGTGACGACCGGCAC	826
	R: CCACACACGGATCGTGGCG	R: AGCGCCATATGCGCCAC	
dnaN	F: ATGAAATTTACCGTTGAACGTGA	F: CCGATTCTCGGTAACCTGCT	833
	R: AATTTCTCATTCGAGAGGATTGC	R: ACGCGACGGTAATCCGGG	
hemD	F: ATGAGTATTCTGATCACCCG	F: GCCTGGAGTTTTCCACTG	666
	R: GAAGCGTTAGTGAGCCGTCTGCG	R: GACCAATAGCCGACAGCGTAG	
hisD	F: GAAACGTTCCATTCCGCGC	F: GTCGGTCTGTATATTCCCGG	894
	R: GCGGATTCCGGCGACCAG	R: GGTAATCGCATCCACCAAATC	
purE	F: GACACCTCAAAAGCAGCGT	F: ACAGGAGTTTTAAGACGCATG	510
	R: AGACGGCGATACCCAGCGG	R: GCAAACTTGCTTCATAGCG	
sucA	F: CGCGCTCAAACAGACCTAC	F: CCGAAGAGAAACGCTGGATC	643
	R: GACGTGGAAAATCGGCGCC	R: GGTTGTTGATAACGATACGTAC	
thrA	F: GTCACGGTGATCGATCCGGT	F: ATCCCGGCCGATCACATGAT	852
	R: CACGATATTGATATTAGCCCG	R: ACCGCCAGCGGCTCCAGCA	

### Clustered regularly interspaced short palindromic repeats (CRISPRs)

11 strains of *Salmonella* Kentucky were further characterized by CRISPRs by two sets of specific primers. CRISPR1 locus was amplified with forward primer A1 (5′-ATAATGCTGCCGTTGGTAA-3′) and reverse primer A2 (5′-TTGATGAGTATGGTGGTTGTGGT-3′). CRISPR2 locus was amplified using primer B1 (5′-CTGTATAAAAGCCTCCCC-3′) and reverse primer B2 (5′-GTTGGTAGAATGTGGTGC-3′). The PCR products were delivered to Sangon Biotech (Shanghai) Co., Ltd. for purification and sequencing. The sequencing results were uploaded to CRISPRfinder (https://crispr.i2bc.paris-saclay.fr/) to obtain the spacers of CRISPR1 and CRISPR2 (Grissa Vergnaud and Pourcel 2008). The directrepeat and spacer names were identified by PasteurCRISPR database for each spacer (http://www.pasteur.fr/recherche/genopole/PF8/crispr/CRISPRDB.html) (Grissa Vergnaud and Pourcel 2007).

## Results

### *Salmonella* prevalence and serotypes

Of the total of 467 samples analyzed, 27 (5.8%) were positive for *Salmonella,* of which the isolation rate from dogs was 7.08% (24/339) and the isolation rate from cats was only 2.31% (3/130)*.* Table 2 shows the detailed prevalence of *Salmonella* in pet from pet hospitals and breeding base; 7 different serotypes were identified among the 27 positive *Salmonella* isolates. These serotypes were *S.* Kentucky, *S*. Typhimurium, *S.* Indiana, *S*. Derby, *S.* Sandiego, *S.* London, *S.* Rissen. The dominant serovar was *S.* Kentucky followed by *S.* Typhimurium, *S.* Indiana and *S.* Derby. At the same time, both *S.* Sandiego and *S*. London found only one sample from a dog, similarly *S.* Rissen found only one strain in a cat sample.

**Table 2:** Serotype distribution of 27 *Salmonella* isolates

Strain no	O antigens	H antigens	Serotype	No. of isolates (%)
S1, S5, S6, S7, S8, S9, S14, S15, S16, S21, S23	8,20	i; z_6_	*S*. Kentucky	11 (40.74%)
S11, S12, S13, S18, S19	1, 4, 5, 12	i; 1, 2	*S*. Typhimurium	5 (18.52%)
S2, S3, S4, S22, T2	1, 4, 12	z; 1, 7	*S*. Indiana	5 (18.52%)
S10, S20, T1	1, 4, 12	f, g	*S*. Derby	3 (11.11%)
S17	1, 4, 5, 12	e, h; e, n, z_15_	*S*. Sandiego	1 (3.70%)
S24	3	l, v; 1, 6	*S*. London	1 (3.70%)
T3	6, 7	f, g	*S*. Rissen	1 (3.70%)

### Multilocus sequence typing (MLST) analysis

**Fig. 1:**
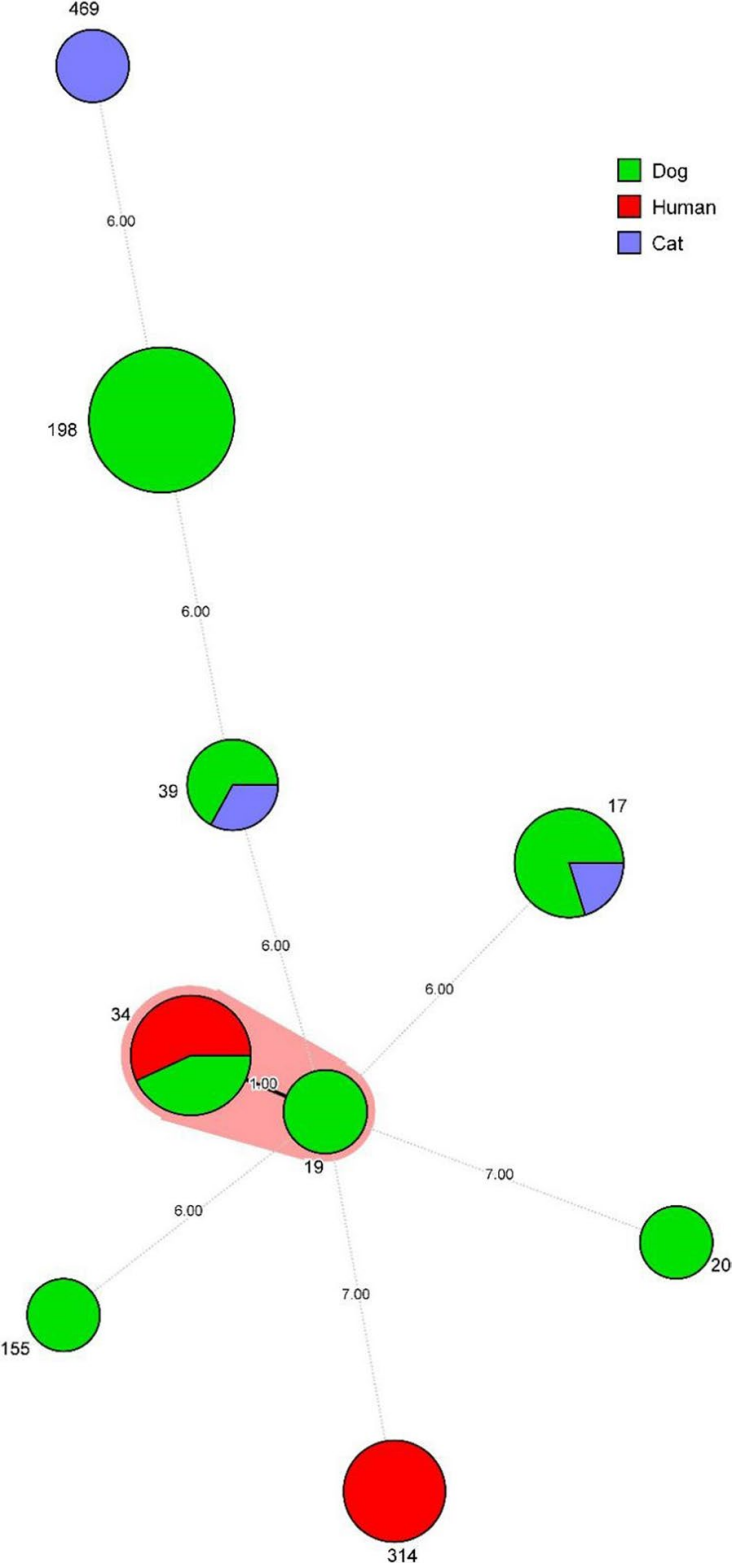
Minimum spanning tree analysis of *Salmonella* Kentucky isolated from cats, dogs and humans. Each circle represents one ST, and the area of the circle corresponds to the number of isolates. Different sources are represented by green, red and light blue respectively

Twenty-seven of the *Salmonella* isolates were amplified and sequenced using seven of housekeeping genes from 399 to 501 bp. Table 2 shows in detail the results of MLST analysis of *Salmonella*, eight different ST patterns were identified among these 27 *Salmonella* isolates. ST198 was the most prevalent STs in the study, represented by 11 (40.74%), followed by ST17 (18.21%), ST34 (11.11%) and ST39 (11.11%). Interestingly, ST17 and ST39 were detected simultaneously in cat and dog samples. At the same time, most of these *Salmonella* isolates came from the same pet hospital. Therefore, *Salmonella* has the risk of cross-infection between pets and can even cause zoonotic diseases. A minimum spanning tree of all STs from the different sources was generated using BioNumerics version 7.6 (Fig. 1).

### Clustered regularly interspaced short palindromic repeats (CRISPRs) analysis

**Fig. 2:**
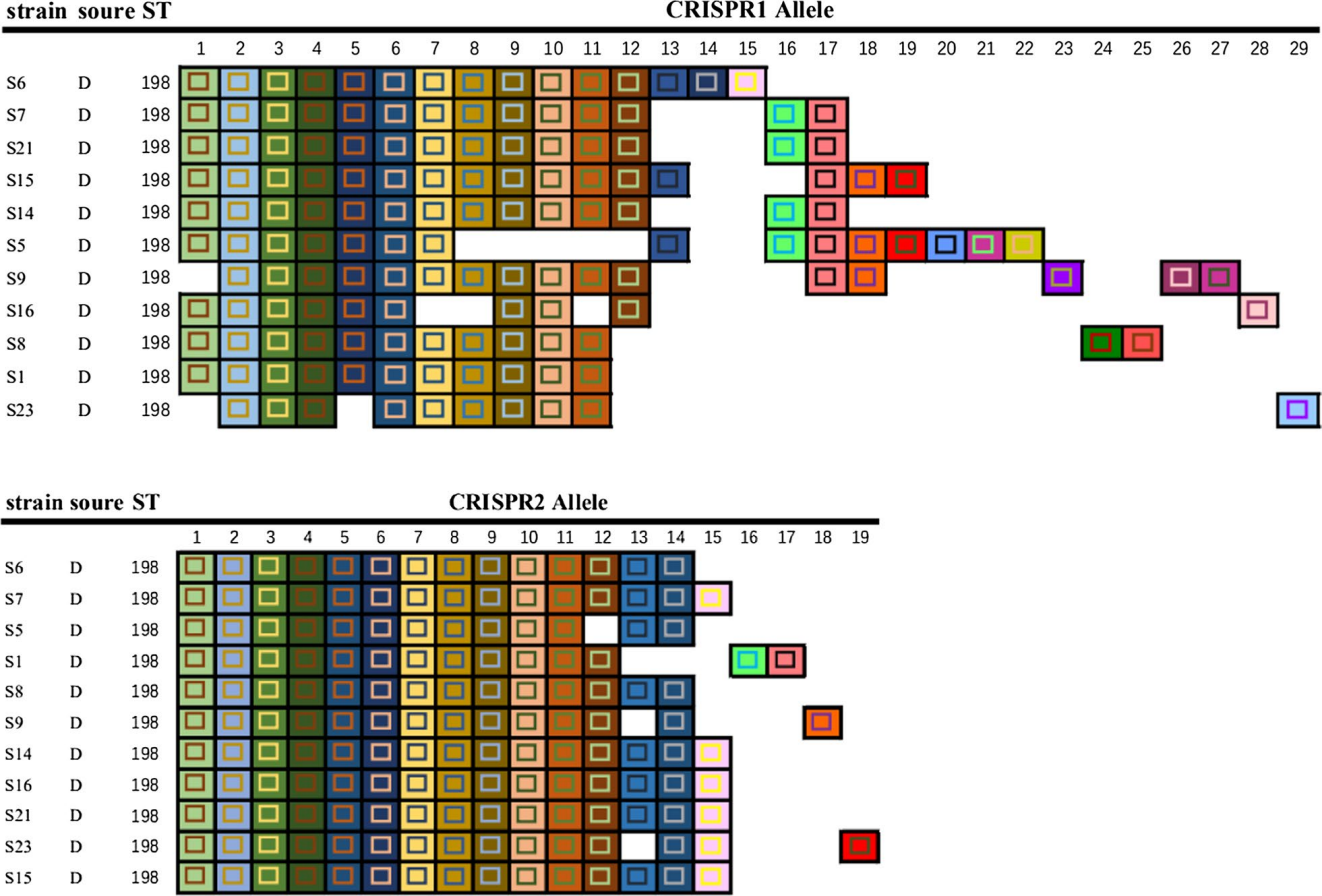
Graphic representation of spacers across the two CRISPR loci for a variety of *Salmonella* Kentucky strains. Repeats are not included; only spacers are represented. Each spacer is represented by a combination of a character in a particular font colour, on a particular background colour. The colour combination allows each spacer to be uniquely represented, whereby squares with similar colour schemes (combination of character colour and background colour) represent identical spacers and different colour combinations represent distinguishable spacers. The spacers are aligned, and the gaps represent the absence of a particular spacer. Above, CRISPR1; Below, CRISPR2

We used CRISPR to investigate the spacer sequences and direct repeat regions of 11 strains of *Salmonella* Kentucky (Table 3). Forty-eight different spacers were discovered, all spacers were 29–35 bp long. Among them, 32 bp (39/48) is dominant. CRISPR1 accounted for 29 and CRISPR2 accounted for 19. Eight new CRISPR spacers were found in CRISPR1 region and four new spacers were found in CRISPR2 loci respectively (Table 4). Figure 2 carefully shows the *S.*Kentucky different spacers arrangements between STs in CRISPR1 and CRISPR2. In CRISPR1, the number of spacers ranged from 10 to 16; for CRISPR2, the number of spacers ranged from 13 to 15. An interlinked dataset of different spacers revealed 9 KCTs among the 11 isolates. Among them, S7, S14 from the same pet hospital were pointed out to belong to KCT2. This result indicates that *Salmonella* has a strong cross infection in the same area. On the other hand, KCT1, KCT2, KCT3, KCT5, and KCT7 are all from the same pet hospital. This result indicates that there is a phenomenon of microevolution in the CRISPR loci in the same serotype. The tested *Salmonella* Kentucky strains has a 29 bp identical highly conserved sequence 5′-CGGTTTATCCCCGCTGGCGCGGGGAACAC-3′ in the conserved region in all CRISPR loci, consistent with the result published by Fabre (Fabre et al. 2012).

**Table 3:** *Salmonella* Kentucky strains used for molecular analysis in this study

Source	Strain (KCTs)	ST
A	S6 (1), S7 (2), S8 (7), S14 (2), S15 (3), S9 (5), S16 (6)	198
B	S1 (8), S5 (4)	198
F	S21 (2)	198
G	S23 (9)	198

A, B, F, G: Different pet hospitals

KCTs: Kentucky CRISPR Types

**Table 4:** *Salmonella* Kentucky spacer name

CRISPR locus	Spacer name^a^ (position)	Spacer DNA sequence (5′-3′)
1	Alt1 (1)	AAAAACTAAATCGCAAATAACCAAAAATATCA
1	Conc15 (2)	TGGCGATAATGTACGACGAGTTGCCAAAATAT
1	Ana3 (3)	ACCAAAACACTGAAAGCCATTCCTCTGGCGTA
1	Ber1 (4)	TGACGGACAACGCGGCCAGTTAGTTTTACCTC
1	Ken17 (5)	CGCAGCGAAGCGAGGACATAAAAGCATTACGG
1	Ken18 (6)	CTGCGCCAGTGCGGTGATATCGTTGCTCATAG
1	Ken19 (7)	GCCTACATAGCCGTAAAAGTCGTCCTGACTAA
1	Ken20 (8)	TTAATACTGTCAACCAGCGCCCCCTGATTGTC
1	Ken37 (9)	TTCGATATGAACGACGCGCAAGCAAAAGAATT
1	Ken38 (10)	TTGCAGGCCCTGGTAGTTGACGCTATCGGGGC
1	Ken39 (11)	GGCGTTCTGGTTCTGGCGGCTAAGATAAAGGG
1	Newp8 (12)	GGGATCAGCACCGACAACCTAAAACCACTGTT
1	Chin1var1 (13)	GTTATGAGTTTTGAGCGTTTTGTGCCGTCGCCC
1	Ken21* (14)	GCAGCGGCGATACGTGTAAAAATCCCCTGGT
1	Ken22* (15)	ATGTGACGATCTGCGGTGGTTACCAGCCAACA
1	Ken40 (16)	GCAGCGGCGATACGTGGAAAAAACCCCCTGGT
1	Ken41 (17)	AGGTGACGATCTGCGGTGGTTACCAGCCAACA
1	Ken42 (18)	GTTATGAGTTTTGAGCGTTTTGTGCCGTCGCC
1	Ken50 (19)	TGAGTCTCTATGGTTAGCAATAATGCGCACAT
1	Ken51 (20)	CTCTCCAGTTCGGCCAGAAACTCCCTGGTCTG
1	Ken52 (21)	GCGCTTTAACGCCCGCTCGCCCCTGAACCCCT
1	Ken53 (22)	CTCCTCGCATTTGTGACTTTCTGGATCATCGG
1	Ken40var1 (23)	GCAGCGGCGATACGTGGAAAAACCCCCCTGGT
1	Ken23* (24)	GGGATCAACACCGACAAACCTAAAACCACTGTT
1	Ken24* (25)	GCATCGGCGATATGTGGAAAAAACCCCCTGGTCG
1	Ken25* (26)	AAAAACTAAATAAGCAAATAACCAAAAATATCA
1	Ken26* (27)	TGAGTCTCTATGGTTAGCAATAATGCGCACATC
1	Ken27* (28)	GGCGTTCTGGTTCTGGCGGCTAAGAAAAGGCCG
1	Ken28* (29)	GTGGATCATTACCGACATCCTAAAACCACTGTTTC
2	EntB0 (1)	GGCTACACGCAAAAATTCCAGTCGTTGGCGCA
2	MonB22 (2)	CCAGCCTCCCGTTACGCATATTTGAGGGTTTT
2	MonB24 (3)	GCGGCGCTGATTATGATTTCCCGATAATTTAT
2	NiaB2 (4)	CTTTCGGGTTGAAAGCAGTAGCACTCATGCCC
2	KenB29 (5)	CATTCCGGCGAGACTGAGCAGCGGGCGAACCG
2	ParcB2 (6)	GGCCGTCTGCATTGAGCGCAGTTTTAACGCGT
2	KenB30 (7)	TACGGATGTGTGACGGAGTCGCGTTTATGGCG
2	KenB31 (8)	ACCGTTTCACGGATGAGAGTTGGCGGAAGCGC
2	KenB32 (9)	CAGGAATAGACCTCTTTTGCGCTGTGGCTTGC
2	CholB4 (10)	TGCGCCACGCCTATACCATCCGAGTTTTGAGC
2	BloB2 (11)	AACAAACTGGAGCGAAATTTCGCACATGCCCC
2	KenB33 (12)	GTGCGGTTTCTTGCCGAATAGTGCCTCGTACT
2	Tho6 (13)	TTCAGAGAATTGCCGCCAATAGAACAGCGCAA
2	KenB42 (14)	AGTCTTTCAGTCCATTGGTCTATGATCTCTCG
2	KenB43 (15)	ATGATGTATCGGCCATCGCGCCGGGTTATGTT
2	KenB28* (16)	AACCAAACTGGAGCGAAATTTCGCACATGCCCCC
2	KenB33* (17)	TTCAGAGAATTGCCGCCCATAGACAACCGCAA
2	KenB34* (18)	ATGATGTATTCGGCATCGCGCCGGGTTATGTG
2	KenB35* (19)	GGCTTCGTGCGCCGCACATCGCAGCGACT

^a^An asterisk shows a novel spacer identified in the current study

## Discussing

In this study, *Salmonella* was isolated from pet dogs and cats in Xuzhou City, Jiangsu Province, China. The isolation rates were 7.08% and 2.31%, respectively. The prevalence of *Salmonella* in dogs was similar in parts of Japan (Yukawa et al. 2019). However, the prevalence was lower than Ethiopia, Canada and Thailand (Kiflu et al. 2017; Leonard et al. 2011; Wu et al. 2020). On the other hand, the prevalence of *Salmonella* in pet cats was lower than that of dogs and humans, which was consistent with the findings of South Africa and Iraq (Zenadal-Obaidi and Al-Talibi 2014; GelawNthaba and Matle 2018). Worryingly, we found that *Salmonella* is more likely to infect older or young pets. This result is the same as the previous study (Reimschuessel et al. 2017; Jay-Russell et al. 2014; Tupler et al. 2012). In fact, some factors need to be considered when we analyze the prevalence of *Salmonella* in different regions, including pet hospital management, breeding condition, sampling method, sampling season and isolation method (Seepersadsingh Adesiyun and Seebaransingh 2004). In general, *Salmonella* will pose a great threat to human public health, especially for children and the elderly. For serotypes, the main widespread were *Salmonella* Kentucky and *Salmonella* Typhimurium in this study. Therefore, we analyze *Salmonella* with different molecular methods to determine its clonal structure and determining the diversity among STs in the same serovar.

*Salmonella* Kentucky has a wide range of prevalence and many hosts and through experiments, it has been found that compared with other serotypes, its drug resistance was generally stronger (Zankari et al. 2013). With the rapid development of molecular biology, the typing methods of *Salmonella* were gradually diversified. MLST and CRISPR molecular typing were commonly used typing methods in recent years. Combined with PCR technology, special bacterial gene fragments were amplified and sequenced. Through sequence comparison software, we can compare the differences of strains at the genetic level and understand the genetic relationship between strains. The resolution of the two methods were higher than that of traditional serotyping. MLST molecular typing relies on multiple conservative housekeeping gene sequences, with low sequence variation, good experimental reproducibility, and reliable results, which can distinguish the same serotypes (Ho et al. 2017); while CRISPR molecular typing relies on the CRISPR loci of bacteria spacer sequence, the spacer sequence was a short DNA obtained from foreign nucleic acids, such as phage or plasmids, which were inserted into the bacterial chromosomes to prevent them from being infected by plasmids or homologous phages (Barrangou et al. 2007). Due to the diversity of phages and plasmids pools in the environment, different CRISPRs had emerged. Thus, CRISPRs had higher resolution in differentiate outbreak strains/clones in epidemic clones (Liu et al. 2011). The deletion and insertion of the spacer sequence form a high degree of polymorphism of the sequence, which makes the CRISPR typing diversified. This method had high resolution to distinguish between strains with close relationships.

MLST was usually used to distinguish different *Salmonella* subtypes, and it can break through the limitations of traditional serotyping. We used MLST to perform rapid and accurate identification of serotyping of pet *Salmonella* from different sources. Each strain has been profiled by different seven alleles. The results showed that a total eight different STs were identified in 27 isolates, including ST198, ST17, ST34, ST19, ST39, ST20, ST469 and ST155. Among them, ST198 was the most frequent genotype recovered in this study. *Salmonella* Kentucky was found in many countries, such as the United States, Denmark, Britain, France and China, and its hosts mainly include poultry, pets and humans. Although both ST198 and ST314 in this study correspond to *Salmonella* Kentucky, but their seven pairs of housekeeping genes were not consistent. Le Hello et al. performed MLST typing on 66 isolates and obtained 9 ST types, ST198 was the dominant type, accounting for 74%, ST314 was also detected, but the proportion was relatively low (Le Hello et al. 2011). Similarly, ST34 and ST19 are closely related. There was only one housekeeping gene (*dna*N) between ST34 and ST19 that has been mutated. Previous experiments have shown that ST34 was more resistant than ST19 (Zankari et al. 2013), and both belong to *Salmonella* typhimurium. ST17 was the more common ST type of *Salmonella* from chickens in Jiangsu Province. In recent years, human infections caused by S*almonella* Indiana have also increased (Gong et al. 2016). Finally, ST39, ST469, and ST155 appeared multiple times in animal-derived and food-borne *Salmonella* (Gong et al. 2016; Yang et al. 2016; Zhou et al. 2017). The ST type appearing in this study was widely present in all kinds of animals and animal products. It was very likely that pet dogs and cats have been in contact with other animals infected by *Salmonella* or eat contaminated food, and the infected pets may spread *Salmonella* to humans and cause outbreaks of salmonellosis through close contact with humans.

CRISPR typing was performed on 11 strains of *Salmonella* Kentucky, and the CRISPR1 and CRISPR2 loci of the strains were amplified and sequenced. By searching the CRISPRFinder database, we found the repeat sequences and spacer sequences, and draw sequence maps. The results showed that the fixed repeat sequences of the two loci were the same. There were 29 kinds of spacer sequences at the CRISPR1 loci, and 8 kinds of new spacer sequences have emerged; there were 19 kinds of spacer sequences at the CRISPR2 loci, and 4 new spacer sequences had appeared. Figure 2 showed that the spacers of CRISPR1 and CRISPR2 were diversity in *S.*Kentucky. The reason for this phenomenon was due to the duplication of a single spacer-DR units or the deletion of single or multiple spacer-DR units. The results of Fabre et al. showed that the number of CRISPR1 spacers in *Salmonella* Kentucky was 19 or 18, and the number of CRISPR2 spacers was 18 or 17 (Gong et al. 2016). In this article, most of the *Salmonella* Kentucky strains had repeat sequence deletions and insertions at both CRISPR loci, this phenomenon is called CRISPRS microevolution. In other words, the change process of the spacer loci was considered to be the evolution process of *Salmonella*, the dynamic changed of the spacer sequence in the locus can not only cause short-term phenotype changes, but also cause long-term subclass evolution (Fricke et al. 2011; Horvath et al. 2008). *Salmonella* Kentucky CRISPR typing (KCT) identified nine KCTs among the 27 isolates from pet dogs. This was consistent with the results of a previous study that CRISPR typing has a better ability to distinguish *Salmonella* Kentucky than MLST (Fen et al. 2011). It was found that multiple KCTs were separated in the same pet hospital. This diversity should be attributed to the sanitation and management conditions of the pet hospital.

According to the 11 strains of *Salmonella* Kentucky in this study were all ST198, CRISPR molecular typing divides the strains of the same ST type into multiple types. This proved that CRISPR typing is more detailed, not only had the ability to distinguish strains of different serotypes, but also had a certain ability to distinguish strains with high homology, with a higher resolution than MLST typing. However, due to the high variability of the bacterial CRISPR loci sequence and the continuous emergence of new spacer sequences, the CRISPR database had some shortcomings. The experimental data need to be continuously updated and improved by researchers. This was of great significance for data sharing in different countries and regions and for the epidemiological analysis of *Salmonella.*

## Data Availability

Please contact to the authors for all request.
